# Randomized Study of Antithrombin in Early-Onset Preeclampsia: KOUNO-TORI Study

**DOI:** 10.1161/HYPERTENSIONAHA.125.25431

**Published:** 2026-03-06

**Authors:** Jun Takeda, Daisuke Tachibana, Atsuo Itakura, Kenichi Takagi, Shoichi Nakami, Hirotaka Mano, Takao Kobayashi, Naohiro Kanayama, Hiroshi Sameshima, Mamoru Morikawa, Haruhiko Sago, Tomoko Adachi, Akihide Ohkuchi, Satoru Takeda, Hisashi Masuyama, Hiroyuki Seki, Shigeru Saito, on behalf of the KOUNO-TORI Study Group, Soromon Kataoka

**Affiliations:** 1 Department of Obstetrics and Gynecology, Juntendo University Faculty of Medicine, Bunkyo-ku, Japan (J.T., A.I., S.T.).; 2 Department of Obstetrics and Gynecology, Osaka Metropolitan University Graduate School of Medicine, Japan (D.T.).; 3 Clinical Science Department, Development Division (K.T.), Kyowa Kirin Co., Ltd., Chiyoda-ku, Japan.; 4 Clinical Development Center, Development Division (S.N.), Kyowa Kirin Co., Ltd., Chiyoda-ku, Japan.; 5 Biostatistical Science Group, Biometrics Department, Development Division (H.M.), Kyowa Kirin Co., Ltd., Chiyoda-ku, Japan.; 6 Department of Obstetrics and Gynecology, Hamamatsu Medical Center, Japan (T.K.).; 7 Shizuoka College of Medicalcare Science, Hamamatsu, Japan (N.K.).; 8 University of Miyazaki, Japan (H.S.).; 9 Department of Obstetrics and Gynecology, Kansai Medical University, Hirakata, Japan (M.M.).; 10 Sanno Birth Center, Minato-ku, Japan (H.S.).; 11 Imperial Gift Foundation, Aiiku Maternal and Child Health Center, Aiiku Hospital, Minato-ku, Japan (T.A.).; 12 Department of Obstetrics and Gynecology, Jichi Medical University, Shimotsuke, Japan (A.O.).; 13 Department of Obstetrics and Gynecology, Okayama University Hospital, Kita-ku, Japan (H.M.).; 14 Saitama Medical Center, Saitama Medical University, Kawagoe, Japan (H.S.).; 15 University of Toyama, Japan (S.S.).

**Keywords:** antithrombins, clinical trial, hemorrhage, pre-eclampsia, pregnancy

## Abstract

**BACKGROUND:**

In preeclampsia, prolonging pregnancy decreases the risks of fetal morbidity and death. This study aimed to evaluate the efficacy and safety of antithrombin in prolonging pregnancy early-onset severe preeclampsia.

**METHODS:**

This was a randomized, double-blind, placebo-controlled study involving women with early-onset preeclampsia from 61 institutions. Participants developed early-onset severe preeclampsia from 24+0 to 31+6 weeks’ gestation and had ≤100% antithrombin activity. Two groups were created, with random and blinded assignment of the participants 1:1 to a placebo (saline) group (n=92) or a recombinant human antithrombin-gamma (rhAT-gamma) group (n=90). The number of days from treatment initiation to delivery was recorded in each group, as the primary end point.

**RESULTS:**

Pregnancy was prolonged by 13 days (95% CI, 10.4–15.6) in the placebo group and 16.9 days (95% CI, 13.8–20.0) in the rhAT-gamma group (*P*=0.07). Compared with the placebo group, hemorrhage-related adverse events occurred at a 19.0% higher rate in the rhAT-gamma group (mean difference [95% CI, 4.3%–32.7%]), and anemia occurred at a 16.8% higher rate (mean difference [95% CI, 2.0%–30.6%]).

**CONCLUSIONS:**

No significant difference in pregnancy prolongation was found between the placebo and rhAT-gamma participants with early-onset severe preeclampsia. However, compared with the placebo group, the rhAT-gamma group appeared to have higher rates of hemorrhage-related adverse events and anemia.

**REGISTRATION:**

URL: https://jrct.niph.go.jp/en-top; Unique identifier: jRCT2080224912. URL: https://clinicaltrials.gov/; Unique identifier: NCT04182373.

**Novelty and Relevance**

**What Is New?**

This study evaluated antithrombin treatment in pregnancy prolongation and its safety in women with early-onset preeclampsia.

**What Is Relevant?**

Preeclampsia is characterized by persistent high blood pressure in pregnant women or those who have recently given birth. Antithrombin, which regulates blood coagulation and suppresses inflammatory responses, is a potential therapeutic option for preeclampsia.

**Clinical/Pathophysiological Implications?**

Antithrombin, compared with placebo, did not prolong pregnancy and was associated with a higher risk of hemorrhage-related side effects and anemia. Therefore, further attention to safety, including dose adjustment, is needed.

Preeclampsia is a leading cause of maternal, fetal, or neonatal morbidity and mortality, accounting for >500 000 fetal and neonatal deaths and >70 000 maternal deaths worldwide.^1^ Long-term follow-up of women with preeclampsia and their children has shown that preeclampsia is a risk factor for future hypertension not only for the mother but also for the child.^2,3^ Preeclampsia exhibits clinical heterogeneity. In particular, the pathophysiology differs in early- and late-onset preeclampsia (onset at <34 or ≥34 weeks of pregnancy, respectively).^4–7^ Early-onset preeclampsia is an immunologic maladaptation resulting in poor placentation and is associated with a high rate of fetal growth restriction. In contrast, late-onset preeclampsia is related primarily to maternal factors, such as metabolic syndrome.^8–10^

Although aspirin effectively prevents the onset of preterm preeclampsia,^11–13^ the fundamental treatment is pregnancy discontinuation. However, earlier delivery carries a higher risk of fetal morbidity and mortality^14^; therefore, it is crucial to prolong the pregnancy as much as possible while ensuring the health of the mother and fetus. Treatment includes hospitalized management of maternal blood pressure and fetal growth, as well as the administration of antihypertensive drugs, such as labetalol, hydralazine, nifedipine, and methyldopa, and magnesium sulfate;^1,15,16^ however, these are symptomatic treatments. Importantly, antihypertensive drugs may reduce uteroplacental blood flow when used excessively.^17–19^ Thus, effective and safe fundamental treatment options are urgently needed.

Proteome- and transcriptome-wide genetic analyses and protein interaction mapping of candidate proteins identified diverse biological pathways potentially underlying the development of preeclampsia, shared through natriuretic peptide signaling, blood pressure regulation, immune tolerance, and thrombin activity regulation in preeclampsia,^20^ supporting that antagonizing thrombin activity by the plasma protein AT (antithrombin) may be a potential therapeutic option for preeclampsia. AT inactivates thrombin and coagulation factors, thereby regulating blood coagulation. pAT (plasma-derived AT) and rhAT-gamma (recombinant human antithrombin-gamma) are used to treat acquired or hereditary AT deficiency or disseminated intravascular coagulation; AT also has antiinflammatory activity.^21^ Furthermore, thrombin enhances soluble sFlt-1 (fms-like tyrosine kinase-1) biosynthesis and release from decidual cells during the first trimester.^22^ Thus, AT may improve uteroplacental perfusion and suppress inflammatory responses, prolonging pregnancy in patients with preeclampsia. Indeed, lower AT activity at diagnosis of early-onset preeclampsia predicts a shorter pregnancy.^23^

Several studies have evaluated the efficacy of AT in patients with early-onset severe preeclampsia.^24–27^ However, the results are conflicting,^28^ and the efficacy remains unclear. We reanalyzed data from the Anthrobin P study^24^ and found that AT prolonged the gestational period in patients with severe preeclampsia with AT activity ≤100%.^29^ We hypothesized that rhAT-gamma prolongs pregnancy in patients with early-onset severe preeclampsia with AT activity ≤100% and evaluated the efficacy and safety of rhAT-gamma in these patients.

## Methods

### Data Availability

Because of the sensitive nature of the data collected for this study, requests to access the data set from other researchers may be sent to Vivli at https://vivli.org/ourmember/kyowa-kirin/. The authors confirm that 1 author with full access to all study data takes responsibility for their integrity and the data analysis. All essential research materials in the Methods, including the full study protocol and statistical analysis plan, can be found in a public registry listed in the Major Resources Table in the Supplemental Material.

### Study Design

The KOUNO-TORI study (KW-3357 Randomized, Multicenter, Double-blind, Placebo-Controlled Phase III Study in Patients With Early-Onset Preeclampsia; Kouno-tori means stork in Japanese) was conducted to test the efficacy and safety of rhAT-gamma (KW-3357; Kyowa Kirin Co., Ltd, Tokyo, Japan) in patients with early-onset severe preeclampsia.^29^ The study protocol was reviewed and first approved by the institutional review board on September 3, 2019, and registered on October 10, 2019 (NCT04182373) and on October 11, 2019 (jRCT2080224912). The study was conducted in accordance with the Declaration of Helsinki and Japanese clinical trial regulations.

**Figure 1.**
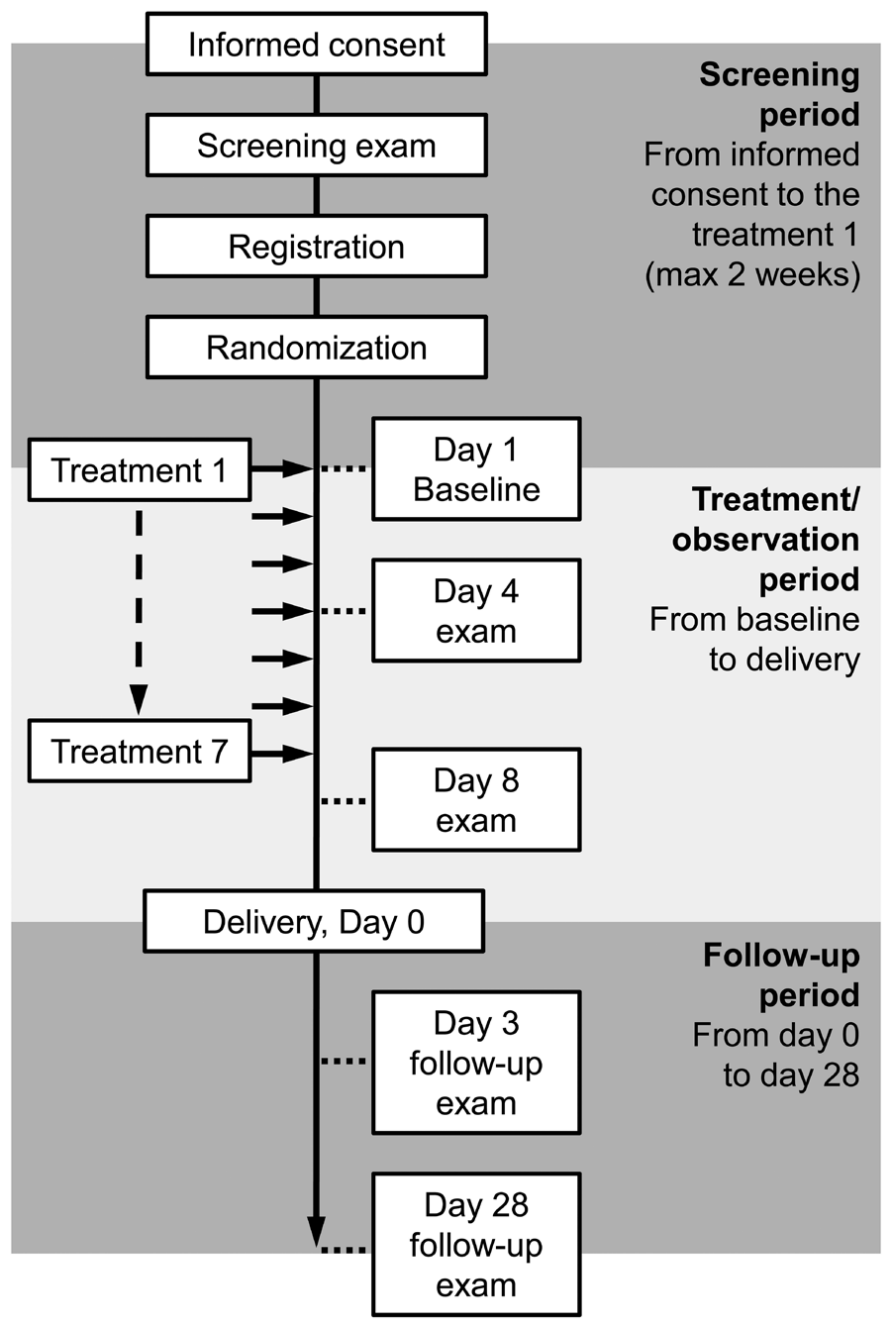
**Schematic diagram of the study timeline.** max indicates maximum.

Patient information was recorded in an electronic case report form at each institution and sent in encrypted form to the central electronic data collection system. Figure 1 shows the study timeline. The protocol has been previously described in detail.^29^

When death, other serious adverse events (AEs), or treatment-emergent AEs were observed, the incidence was reported to the data and safety monitoring committee. The committee evaluated each incident independently and determined whether termination of the study drug or any protocol amendment would be required.

### Patient Selection

Eligible patients were legally adult women (≥20 years old, or ≥18 years old as of a legal amendment in April 2022) at ≥24 and <32 weeks’ gestation at registration who had been diagnosed with early-onset severe preeclampsia with ≤100% AT activity at screening. Preeclampsia and its severity were determined according to the guidelines of the Japan Society for the Study of Hypertension in Pregnancy (Supplemental Methods).^15,29^ Patients with superimposed preeclampsia or chronic hypertension were not included in the study. Fetal growth restriction was clinically diagnosed according to the definition of the Japanese Society of Ultrasonics in Medicine and the diagnosis was made when the estimated fetal body weight fell below the mean weight minus 1.5 SD for the gestational age based on the Japanese fetal growth curve developed by Okai^30,31^ in the absence of chromosomal abnormalities or malformation syndrome. Written consent was obtained from all patients before screening, with the exception of patients in whom AT values were measured within 3 days before the first administration of rhAT-gamma, in which case written consent for use of the data for this study was obtained after AT activity testing. Patients with severe comorbidities, including maternal and fetal indications for early delivery listed in Table S1, were excluded from the study. Using an internet-based interactive web response system, the patients were registered sequentially and dynamically allocated to rhAT-gamma or placebo groups by the interactive web response system at a 1:1 ratio based on AT activity (≤80% or >80%), gestational age (<28 or ≥28 weeks), and proteinuria (present or absent) at screening. Allocation results were stored securely in the interactive web response system.

### Intervention

rhAT-gamma (1800 IU/vial) was dissolved in 36 mL of water and added at 72 IU/kg to 100–150 mL of saline in an infusion bag. Saline was used as placebo. Infusion bags were prepared by preregistered unblinded personnel and distributed to blinded personnel. The patients received intravenous infusion once daily for 7 days (Days 1–7), which was chosen because the Anthrobin P study^13^ demonstrated the safety and efficacy of pAT at 3000 IU/d for 7 days in pregnant women with severe preeclampsia. Treatment was discontinued early by delivery, patients’ withdrawal, or physicians’ decision.

Patients and fetuses were managed and monitored at each site in accordance with Japanese guidelines.^15,31,32^ During the study, corticosteroid use was restricted and long-term use was prohibited. Initiation, dose adjustment, and discontinuation of antihypertensive drugs were unrestricted. Early delivery was carefully determined by physicians at each participating institution referencing the list of maternal and fetal indications for early delivery (Table S1). The above information was written in the study protocol and distributed to all participating institutions. All participating physicians were required to follow the protocol.

### Efficacy and Safety End Points

The primary end point was pregnancy prolongation (days) from treatment initiation to delivery. The main secondary end points were the proportions of women who achieved 28 (for patients at <28 weeks’ gestation at baseline), 32, or 34 weeks’ pregnancy and AT activity transition. Other end points have been described elsewhere.^29^

Safety end points were AEs, drug-related treatment-emergent AEs, laboratory test results, vital signs, and anti-rhAT-gamma antibodies. Hemorrhage-related AEs were evaluated as AEs of special interest.

Plasma AT activity and biomarkers were measured centrally at SRL, Inc (Tokyo, Japan), and anti-rhAT-gamma antibodies at SNBL, Ltd (Kagoshima, Japan), with blinded personnel unable to obtain these values.

### Sample Size

Based on data from the Anthrobin P study,^24^ the pregnancy prolongation period for each group was assumed as follows. For the control group, the distribution of pregnancy prolongation period followed a Weibull distribution with a scale parameter of 10.3552 and a shape parameter of 0.9238, indicating that ≈50% of subjects maintained pregnancy for 7 days after treatment initiation. For the rhAT-gamma group, the pregnancy prolongation period also followed a Weibull distribution, with a scale parameter of 19.0943 and a shape parameter of 1.1494, showing that ≈50% of subjects maintained pregnancy for 14 days after treatment initiation. In both groups, ≈3% of patients maintained pregnancy for 70 days or longer.

The required sample size was determined through simulation (10 000 iterations) to achieve 90% statistical power for detecting a difference between treatment groups using the generalized Wilcoxon test, at a 2-sided significance level of 5%. The resultant sample size was calculated to be a total of 180 subjects, with 90 subjects assigned to each group.^29^

### Statistics

The full analysis set (FAS) and the safety analysis set (SAS) were defined as all randomized patients excluding subjects who did not receive rhAT-gamma. The per-protocol set was defined as the FAS excluding subjects who had significant protocol deviation, which might affect efficacy evaluation. All tabulations, graphical presentations, and statistical analyses were performed using SAS, version 9.4 or higher.

Efficacy was analyzed in the FAS. The pregnancy prolongation period was compared between the rhAT-gamma and placebo groups using the generalized Wilcoxon test. The mean and its 95% CI were estimated using the Kaplan-Meier method in the FAS and per-protocol set. Differences between the 2 treatment groups in other maternal, fetal, and neonatal efficacy end points were evaluated, and 95% CIs were calculated. The hazard ratio of each stratification factor for days of maintaining pregnancy, and its corresponding 95% CI were estimated using a Cox proportional-hazards model including stratification factors as covariates. Subgroup analyses for number of gestational weeks at registration, AT activity, proteinuria, and severity of hypertension at baseline were performed as above. Ad hoc subgroup analyses of the pregnancy prolongation were performed in patients who had PlGF (placental growth factor) levels ≤100 pg/mL or an sFlt-1/PlGF ratio ≥85.

Safety was analyzed in the SAS. All safety items were summarized descriptively. Risk ratios and their 95% CIs were calculated as a post hoc analysis for hemorrhage-related AEs and anemia. All other items were summarized descriptively.

## Results

### Patients’ Disposition

**Figure 2.**
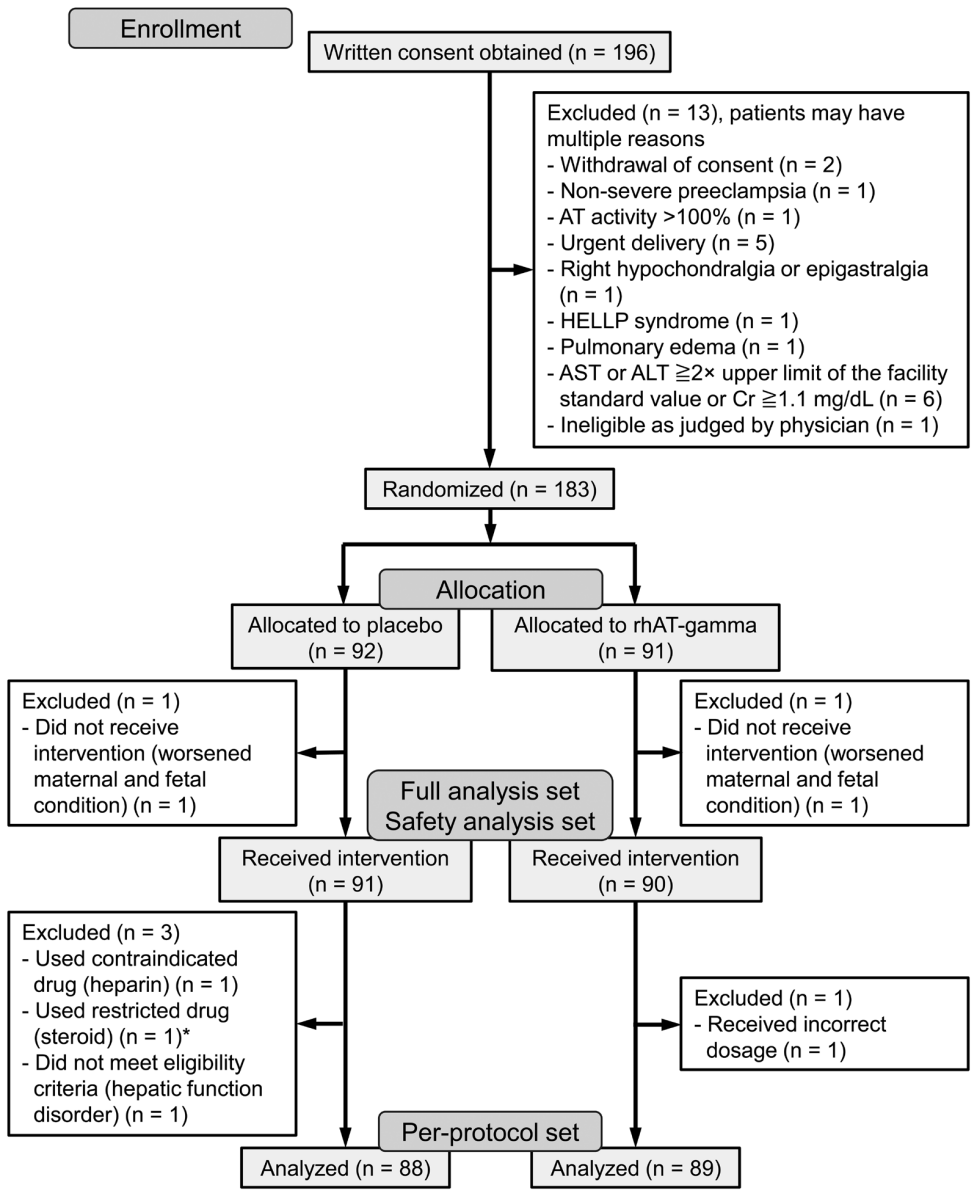
**Patient flowchart.** In this study, 183 patients were randomized; 181 patients (placebo n=91; rhAT-gamma (recombinant human antithrombin-gamma) n=90) comprised the full analysis set and safety analysis set, and 177 patients (placebo n=88; rhAT-gamma n=89) comprised the per-protocol set. *Patients were allowed to use steroids at delivery or in the presence of the following conditions after providing consent before the end of pregnancy: betamethasone 12 mg, twice at a 24-hour interval, intramuscular injection to prevent neonatal respiratory distress syndrome in case premature birth was predicted; topical application onto skin, bronchi, ear, nose, throat, eye, oral cavity, anus, and vagina. Patients with all other types of steroid use were excluded from the per-protocol set. ALT indicates alanine aminotransferase; AST, aspartate aminotransferase; AT, antithrombin; Cr, creatinine; and HELLP, hemolysis, elevated liver enzymes, and low platelets.

From November 19, 2019, 196 patients from 61 institutions were given detailed information about participation in this study. AT activity is readily measured at many institutions in Japan, and patients with AT activity >100% were excluded from recruitment. Among the 196 patients, 13 were excluded before randomization, including 1 patient with AT levels >100%. In total, 183 patients were randomized (placebo n=92; rhAT-gamma n=91; Figure 2). Placebo or rhAT-gamma was administered to 181 patients (placebo n=91; rhAT-gamma n=90; Figure 2; Table S2), all of whom were included in the SAS and FAS. Two patients did not receive the placebo or rhAT-gamma, because of worsened maternal and fetal condition and were excluded from the SAS and FAS. The per-protocol set comprised 177 patients (placebo n=88; rhAT-gamma n=89), excluding patients with critical protocol deviations that could have affected the efficacy analysis. The last patient’s follow-up occurred on June 16, 2023.

The patients’ characteristics (Table 1) were well balanced, with no notable between-group differences. The mean±SD age was 34.6±4.9 years, and the mean±SD baseline gestational age was 29.0±2.2 weeks. The mean ± SD baseline PlGF was 50.1±70.9, and 166/180 patients (placebo n=84; rhAT-gamma n=82) had PlGF ≤100 pg/mL, while 14/180 patients (placebo n=6; rhAT-gamma n=8) had PlGF >100 pg/mL. Patients who had PlGF ≤100 pg/mL had mean ± SD gestation age of 28.8 (2.2) weeks, while patients who had PlGF >100 pg/mL had mean ± SD gestation age of 31.2 (0.9) weeks. Patients who had PlGF ≤100 pg/mL had mean ± SD AT activity of 79.6% (11.6%), while patients who had PlGF >100 pg/mL had the mean ± SD AT activity of 87.7% (34.8%). Notably, 2/91 patients (2.2%, placebo group) and 3/90 patients (3.3%, rhAT-gamma group) discontinued treatment. Reasons for discontinuation were physician’s decision (2/91 patients, 2.2%, placebo group), AE occurrence (1/90 patients, 1.1%, rhAT-gamma group), and patient withdrawal (2/91 patients, 2.2%, rhAT-gamma group).

**Table 1.**
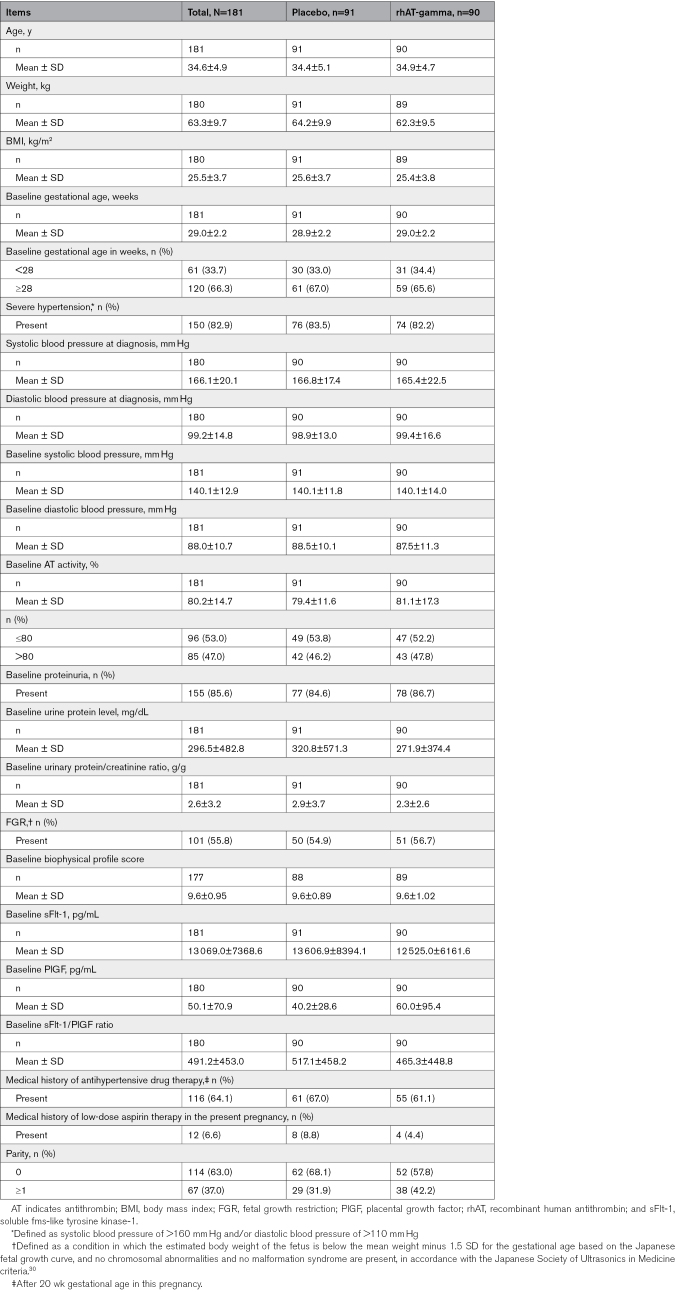
Patients’ Background Information in the Full Analysis Set and Safety Analysis Set

### Prolongation of Pregnancy and Neonatal Body Weight

**Figure 3.**
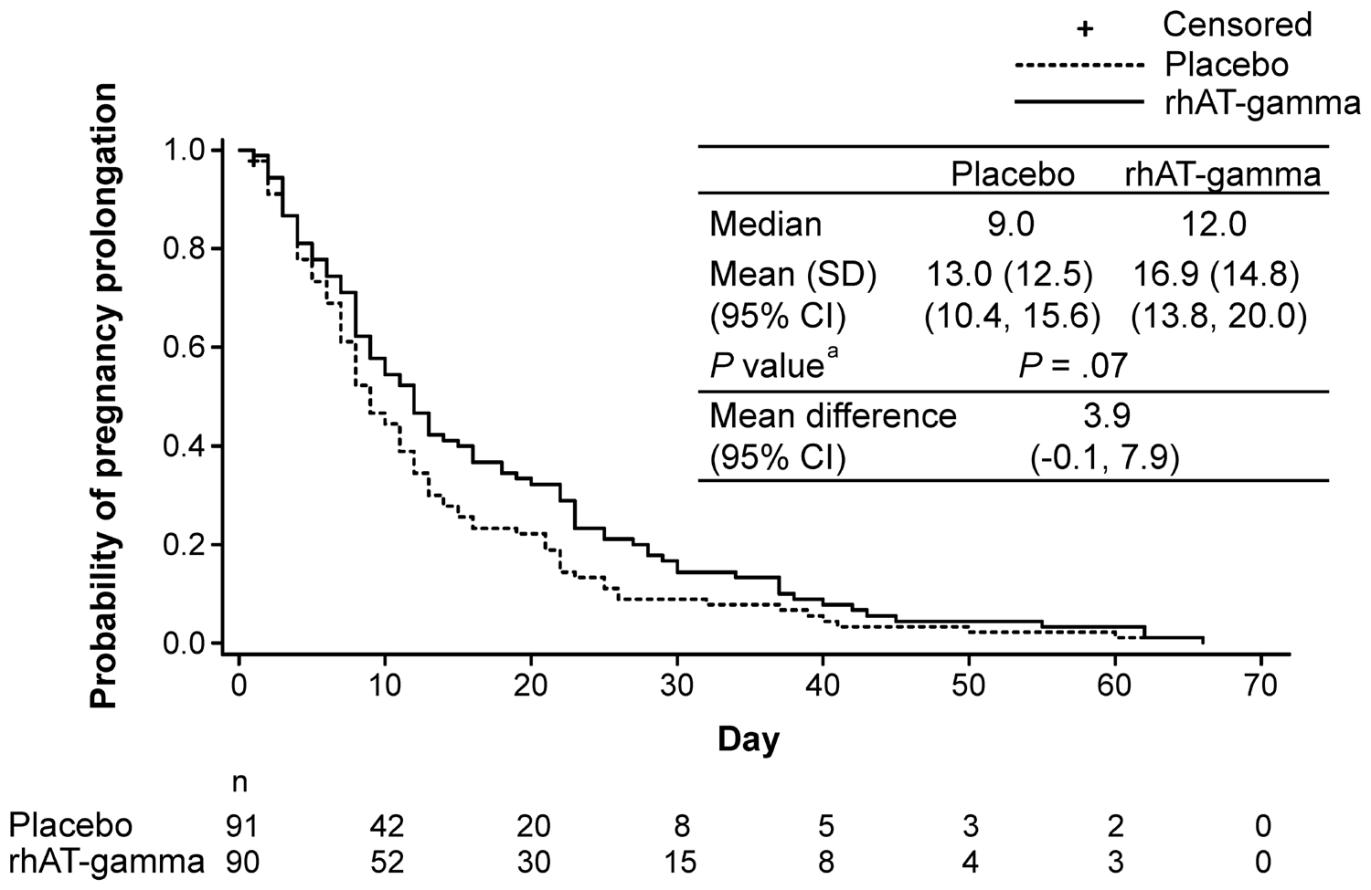
**Kaplan-Meier plot of pregnancy prolongation in full analysis set.** ^a^Generalized Wilcoxon test. Full analysis set (placebo n=91; rhAT-gamma (recombinant human antithrombin-gamma) n=90). The solid line represents the rhAT-gamma group, the dotted line represents the placebo group, and a plus sign represents a censored patient.

The Kaplan-Meier plots for the primary end point (pregnancy prolongation period) were similar between the FAS and per-protocol set (Figure 3 and Figure S1, respectively). During the treatment period, 35/90 (38.9%) patients in the placebo group and 26/90 (28.9%) patients in the rhAT-gamma group underwent medically indicated delivery. The main indications for delivery for all subjects are listed in Table S3.

The mean (95% CI) pregnancy prolongation period was 13.0 (10.4–15.6) days in the placebo group and 16.9 (13.8–20.0) days in the rhAT-gamma group (mean difference, 3.9 days; 95% CI, −0.1 to 7.9 days; *P*=0.07). There was no statistically significant difference in the pregnancy period between the placebo and rhAT-gamma groups (*P*=0.07). The mean ± SD birth weight was 1101.2±397.4 g (placebo group) and 1217.4±504.3 g (rhAT-gamma group; mean difference, 116.2 g [95% CI −17.6 to 250.0 g]; Table S4). The hazard ratio (95% CI) of covariates by Cox regression was 0.774 (0.577–1.040) for the treatment group, 0.670 (0.496–0.906) for AT activity, 1.024 (0.742–1.412) for gestational age, and 0.541 (0.346–0.847) for presence of proteinuria. Baseline AT activity >80% and absence of proteinuria were associated with prolonged pregnancy duration compared with baseline AT activity ≤80% and absence of proteinuria, respectively (Table S5). The ad hoc subgroup analysis showed that the patient subgroup with PlGF levels ≤100 pg/mL had a mean (95% CI) pregnancy prolongation period of 11.8 (9.4–14.2) days in the placebo group (n=84) and 15.3 (12.4–18.2) days in the rhAT-gamma group (n=82; mean difference, 3.4 days [95% CI, −0.3 to 7.2 days]). The patient subgroup with an sFlt-1/PlGF ratio ≥85 had a mean (95% CI) pregnancy prolongation period of 11.4 (9.1–13.7) days in the placebo group (n=84) and 14.3 (11.7–17.0) days in the rhAT-gamma group (n=78; mean difference 2.9 days; 95% CI −0.6 to 6.4 days).

**Figure 4.**
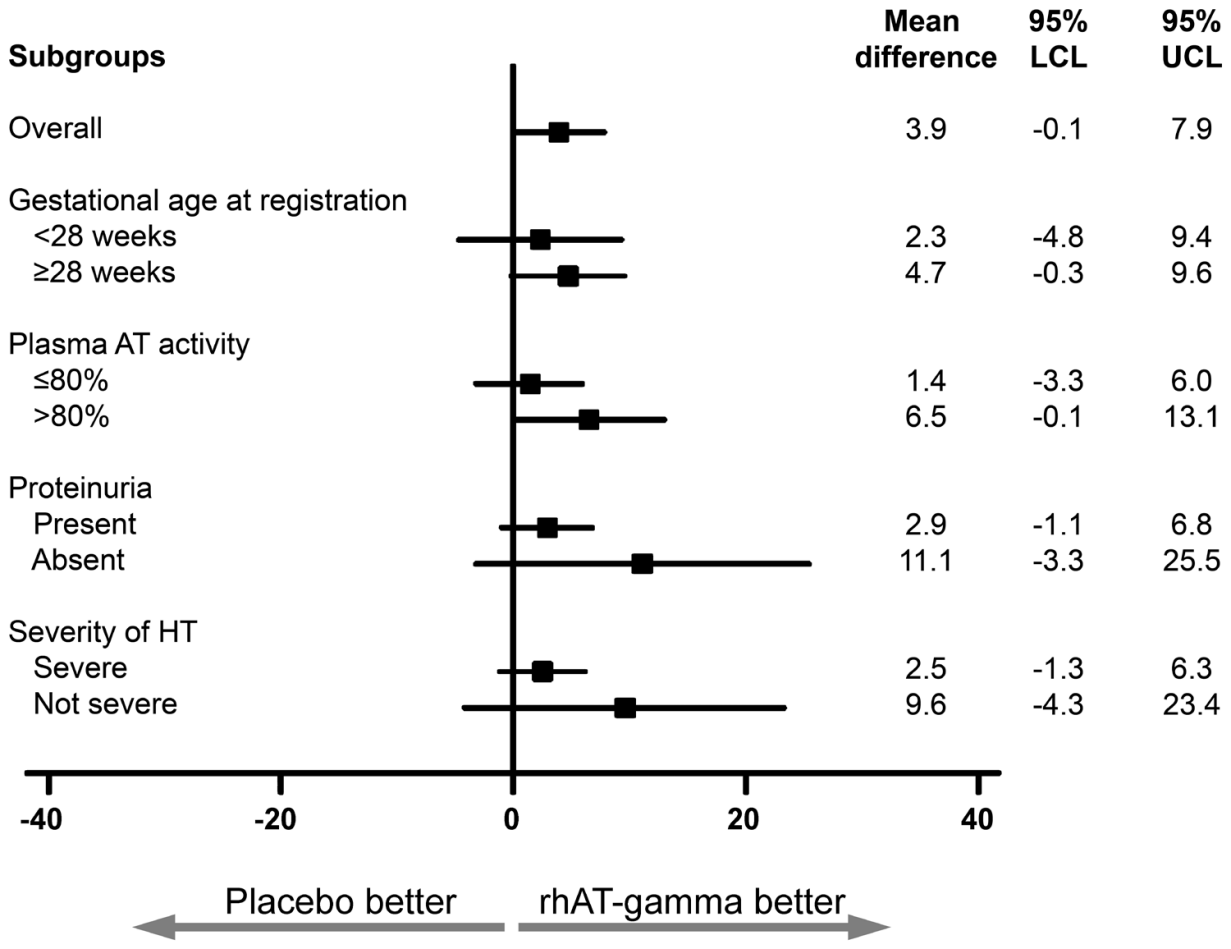
**Forest plot of pregnancy prolongation by subgroups in full analysis set.** The sizes of the subgroups in the placebo and rhAT-gamma (recombinant human antithrombin-gamma) groups were as follows: overall, n=91 and n=90; gestational age at registration <28 weeks, n=30 and n=31; gestational age at registration ≥28 weeks, n=61 and n=59; plasma AT (antithrombin) activity ≤80%, n=49 and n=47; plasma AT activity >80%, n=42 and n=43; proteinuria present, n=77 and n=78; proteinuria absent, n=14 and n=12; severe hypertension (HT), n=76 and n=74; nonsevere HT, n=15 and n=16, respectively. LCL indicates lower confidence level; and UCL, upper confidence level.

The proportions of patients who achieved a gestational age of 28, 32, and 34 weeks did not differ between the groups (Table S6). In the analyses of pregnancy prolongation compared between the rhAT-gamma group and placebo group subdivided based on baseline characteristics, the shift of the point estimates to the right (rhAT-gamma better) was apparently greater in the population with AT activity >80% compared with those with AT activity ≤80%, in the population without proteinuria compared with those with proteinuria, and in the population with nonsevere hypertension compared with those with severe hypertension (Figure 4). In any of these prespecified subgroups, there were no significant differences between the placebo and rhAT-gamma groups (Figure 4).

### Efficacy

AT activity (mean ± SD) increased from 81.1% ± 17.3% (baseline) to 211.5% ± 28.4% (day 8) in the rhAT-gamma group, with no change in the placebo group (79.4% ± 11.6% at baseline, 79.0% ± 11.9% on day 8).

The time course changes of sFlt-1, PlGF, and the sFlt-1/PlGF ratio from baseline to day 8 are summarized in Table S7. There were no notable changes between the 2 treatment groups. Several maternal, fetal, and neonatal efficacy end points, such as duration of hospitalization in a neonatal intensive care unit and duration of respiratory management, are summarized in Table S4. There were no notable differences in maternal, fetal, or neonatal efficacy end points.

### Safety

AEs were observed in 70/91 (76.9%) patients, 28/91 (30.8%) fetuses, and 30/91 (33.0%) neonates in the placebo group and in 69/90 (76.7%) patients, 26/90 (28.9%) fetuses, and 34/90 (37.8%) neonates in the rhAT-gamma group. Hemorrhage-related AEs, considered as AEs of special interest, were observed in 8/91 (8.8%) patients in the placebo group and 25/90 (27.8%) patients in the rhAT-gamma group (mean difference, 19.0% [95% CI, 4.3%–32.7%]; Table 2 and Table S8). The hemorrhage and hematoma incidence rate was higher in the rhAT-gamma versus placebo groups (Table 2). There were no between-group differences in other hemorrhage-related AEs, such as purpura (mean difference, 2.2% [95% CI, −12.3 to 16.7]), disseminated intravascular coagulation, and immune thrombocytopenia (Table S8). Mean blood loss during delivery was 784.7 mL in the placebo group and 806.5 mL in the rhAT-gamma group, and the mean difference (95% CI) between groups was 21.8 mL (−145.0 to 188.7 mL; Table S4). Volumes of blood loss in the placebo group versus in the rhAT-gamma group were ≥1000 mL in 21/90 (23.3%) patients versus 22/89 (24.7%) patients; ≥1500 mL in 6/90 (6.7%) versus 11/89 (12.4%); and ≥2000 mL in 4/90 (4.4%) versus 4/89 (4.5%), respectively. In addition, RBC transfusion was performed in 7/91 (7.7%) patients in the placebo group and 18/90 (20.0%) patients in the rhAT-gamma group. The incidence of anemia was 9/91 (9.9%) patients in the placebo group and 24/90 (26.7%) patients in the rhAT-gamma group, showing a significant mean difference of 16.8% (95% CI, 2.0%–30.6%; Table 2). Drug-related treatment-emergent AEs were observed in 2/91 (2.2%) patients in the placebo group, 7/90 (7.8%) patients in the rhAT-gamma group, none of the fetuses in any group, no newborns in the placebo group, and 1/90 newborns (1.1%) in the rhAT-gamma group (Table S9).

**Table 2.**
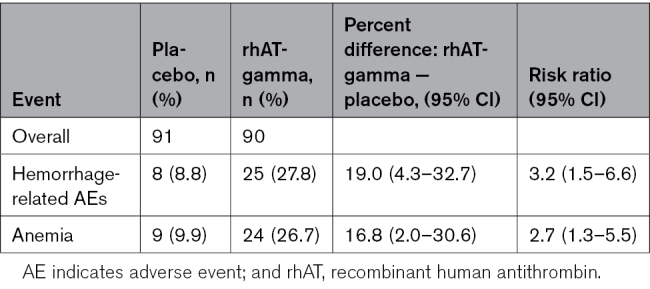
Incidence of Hemorrhage-Related Adverse Events (Adverse Events of Special Interest) and Anemia in Safety Analysis Set

Anti-rhAT-gamma antibody values before and after treatment initiation were obtained from 89 patients in each group. In the rhAT-gamma group, 4.5% of the patients developed anti-rhAT-gamma antibodies, but no anti-rhAT-gamma antibody-associated AEs were observed. There were no notable changes after treatment or between-group differences in other safety evaluation items, such as clinical test values and vital signs.

## Discussion

In this study, rhAT-gamma administration did not prolong pregnancy in patients with early-onset severe preeclampsia, but was associated with a higher risk of hemorrhage-related AEs.

Several randomized controlled studies have evaluated AT therapy for pregnancy prolongation in patients with preeclampsia,^24–27^ with controversial results. Pregnancy was significantly prolonged by pAT versus placebo (Anthrobin P study)^13^ and by high-dose versus standard-dose AT,^25^ whereas no difference was found between rhAT and placebo (PRESERVE-1 study)^26^ or between pAT and placebo (ATIII-EPAS study).^27^

The heterogeneity of the study population may explain these conflicting outcomes. We reanalyzed the data from the Anthrobin P study and found that AT prolonged gestation only in severe cases or in those with AT activity ≤100%. In the PRESERVE-1 study,^26^ which is a randomized, double‑blind, placebo‑controlled trial evaluating rhAT for early‑onset preeclampsia, AT was ineffective, potentially because baseline mean AT activity was as high as 94.70% in the rhAT group and 93.05% in the placebo group, and approximately half of the patients had AT activity >100%. Additionally, both nonsevere preeclampsia and superimposed preeclampsia were included in the study^26^ Furthermore, because of the small sample sizes and low incidence of severe preeclampsia in the AT and placebo groups in the ATIII-EPAS study^27^ (n=20 and n=18; 40.0% and 28.8%, respectively), which is a randomized, double‑blind, placebo‑controlled trial evaluating pAT for early‑onset preeclampsia, its efficacy results for AT treatment in prolonging pregnancy may not have been detectable. In this KOUNO-TORI study, only patients with AT activity ≤100% were enrolled, enabling us to test the efficacy of AT in patients with low baseline AT activity (rhAT-gamma group, 81.1% ± 17.3%; placebo group, 79.4% ± 11.6%). Thus, this is the first report on whether AT treatment prolongs pregnancy in patients with severe preeclampsia and reduced AT activity.

### Clinical Implications

This KOUNO-TORI study did not show overall significant prolongation of pregnancy by rhAT-gamma. Clinical heterogeneity exists in preeclampsia patients, such as presence of fetal growth restriction, number of parities, obesity status, pregnancy after in vitro fertilization, and presence of relatively high blood pressure in the early pregnancy period. In addition, molecular heterogeneity exists in preeclampsia patients also: recent studies using omics technology and systems biology have identified the following types of preeclampsia: placental preeclampsia, metabolic preeclampsia, immunologic preeclampsia, and maternal preeclampsia.^33^ Further subdivision of cases with different clinical or molecular types of preeclampsia, including patients with AT activity <100% or each pathological condition, may reveal which patients may benefit from AT preparations.

For reference, an ad hoc subgroup analysis was conducted using the angiogenic factors sFlt-1 and PlGF. Even in the population potentially diagnosed as having preeclampsia based on angiogenic factors, no significant difference in pregnancy prolongation was observed between the placebo and rhAT-gamma groups.

In the current study, the observed increased risk of hemorrhage-related AEs with rhAT-gamma calls for further attention to safety, including the need for dose adjustment. Hemorrhage-related AEs and anemia were more frequently observed in patients in the rhAT-gamma group and usually occurred at the incision or laceration site. In the rhAT-gamma group, AT activity was ≈211% on day 8. This contrasts with the Anthrobin P study,^24^ wherein it showed a 47.2% increase from the baseline value of 72.3%. Patients in that study received daily infusions of 3000 units of pAT (equivalent to 3600 units of rhAT-gamma) for 7 days. In our study, patients received 72 IU/kg of rhAT-gamma for 7 days because rhAT-gamma was already marketed as a weight-equivalent dose drug, and 72 IU/kg was considered equivalent to 3000 units of pAT. Consequently, the mean ± SD dose of rhAT-gamma was 4454.3±686.34 IU/d. As described above, the administered dose in the current study was higher than that in the Anthrobin P study, which may have caused the increased incidence of hemorrhage-related AEs.

### Research Implications

The efficacy of AT therapy has not been established. The subgroup analyses may suggest that rhAT-gamma might be more effective in pregnancy prolongation in patient populations with mild preeclampsia than severe preeclampsia, although there was no significant difference between the placebo and rhAT-gamma groups in any of these prespecified subgroups. With respect to safety, hemorrhage-related AEs and anemia were more common in the rhAT-gamma group, which was more apparent after delivery (Table S10). However, there was no significant difference between the groups in the proportion of events occurring during pregnancy (Table S10). Notably, no group differences were observed in blood loss during delivery (Table S4). Nonetheless, maternal hemorrhage is a leading cause of maternal morbidity and mortality. Further studies involving AT treatment need to focus on safety by adjusting the dose to the equivalent of 3000 units of pAT to lower the risk of hemorrhage-related AEs and anemia after delivery.

### Strengths and Limitations

Before the current study, data from the Anthrobin P study^24^ were reanalyzed, and the target population was rationally selected.^29^ Therefore, the current study is robust and comprehensive.

A limitation of this study is that the expected pregnancy period at registration and the timing of delivery were subjectively determined by investigators, despite our distribution of procedural documents. The included patients were distributed equally among participating institutions, and the largest number in a single institution was 9; therefore, its influence on our results was considered minor. Finally, because all patients in this study were Japanese, the results may not be generalizable to other races.

### Conclusions

In this study, rhAT-gamma did not prolong pregnancy in patients with early-onset severe preeclampsia compared with placebo. The data suggested that hemorrhage-related AEs and anemia were more common in the rhAT-gamma group.

### Perspectives

Preeclampsia is a leading cause of maternal, fetal, and neonatal morbidity and mortality. Antagonizing thrombin activity using the plasma protein antithrombin is a potential therapeutic option. However, available results remain conflicting, and its efficacy remains unclear. This multicenter, randomized, double-blind, placebo-controlled study evaluated the efficacy and safety of rhAT-gamma in women with early-onset preeclampsia and ≤100% antithrombin activity at screening. Comparing 91 patients in the placebo group with 90 patients in the rhAT-gamma group, we showed that administration of rhAT-gamma did not significantly prolong pregnancy. We found that rhAT-gamma was associated with a higher risk of hemorrhage-related AEs and anemia compared with placebo. It should be noted that the results may not be generalizable to other populations because all patients in this study were Japanese. Despite this limitation, this study adds to the growing body of evidence on the efficacy and safety of antithrombin in the treatment of preeclampsia and provides a foundation for future research.

## Article Information

### Acknowledgments

The authors thank Yuji Tanaka (Statistical Programming & Data Operations Group, Biometrics Department, Development Division, Kyowa Kirin Co., Ltd) for his support in the statistical analyses. This study was financially supported by Kyowa Kirin Co., Ltd, and the Japan Blood Product Organization, including medical writing (writer: M. Tauchi, PhD) and English editing through ASCA Corporation, Osaka, Japan (https://www.asca-co.com/english_site/index.html).

### Author Contributions

J. Takeda: Investigation, Resources, Writing—Original Draft, Writing—Reviewing and Editing. D. Tachibana: Investigation, Resources, Writing—Reviewing and Editing. A. Itakura: Investigation, Resources, Writing—Reviewing and Editing. K. Takagi: Conceptualization, Methodology, Writing—Original Draft, Writing—Reviewing and Editing, Visualization, Project administration. S. Nakami: Conceptualization, Methodology, Writing—Original Draft, Writing—Reviewing and Editing, Visualization, Project administration. H. Mano: Formal analysis, Conceptualization, Methodology, Writing—Original draft, Writing—Reviewing and Editing. T. Kobayashi: Conceptualization, Methodology, Writing—Reviewing and Editing, Supervision. N. Kanayama: Conceptualization, Methodology, Writing—Reviewing and Editing, Supervision. H. Sameshima: Conceptualization, Methodology, Writing—Reviewing and Editing, Supervision. M. Morikawa: Investigation, Resources, Conceptualization, Methodology, Writing—Reviewing and Editing, Supervision. H. Sago: Investigation, Resources, Conceptualization, Methodology, Writing—Reviewing and Editing, Supervision. T. Adachi: Conceptualization, Methodology, Writing—Reviewing and Editing, Supervision. A. Ohkuchi: Investigation, Resources, Conceptualization, Methodology, Writing—Reviewing and Editing, Supervision. S. Takeda: Conceptualization, Methodology, Writing—Reviewing and Editing, Supervision. H. Masuyama: Investigation, Resources, Conceptualization, Methodology, Writing—Reviewing and Editing, Supervision. H. Seki: Investigation, Resources, Conceptualization, Methodology, Writing—Reviewing and Editing, Supervision, Project administration. S. Saito: Conceptualization, Methodology, Writing—Reviewing and Editing, Supervision, Project administration.

### Disclosures

J. Takeda, D. Tachibana, A. Itakura, T. Kobayashi, N. Kanayama, H. Sameshima, M. Morikawa, H. Sago, T. Adachi, A. Ohkuchi, S. Takeda, H. Masuyama, H. Seki, and S. Saito received research funding for the present article from Kyowa Kirin Co., Ltd. H. Seki received consulting fees from StemCell Institute, Inc. S. Saito received consulting fees from Kyowa Kirin Co., Ltd, payment or honoraria for lectures, presentations, speakers’ bureaus, article writing or educational events from Astellas Pharma, Inc, Alexion Pharmaceuticals, Inc, Japan Blood Products Organization, Kyowa Kirin Co., Ltd, UCB Japan Co. Ltd, Asahi Kasei Corporation, Shino-Test Corporation, Kaken Pharmaceutical Co., Ltd, Otsuka Pharmaceutical Co., Ltd, Roche Diagnostics K.K., TOA Biopharma Co., Ltd, and Tsumura & Co., payment for expert testimony from Kyowa Kirin Co., Ltd, Roche Diagnostics K.K., and Japan Blood Products Organization, and served in a leadership or fiduciary role in Japan Society of Perinatal and Neonatal Medicine.

K. Takagi, S. Nakami, and H. Mano are employees of Kyowa Kirin Co., Ltd.

### Supplemental Material

Methods

Tables S1–S10

Figure S1

Major Resources Table

References

## Supplementary Material
