# Supplementary material for: Randomized Study of Antithrombin in Early-Onset Preeclampsia: KOUNO-TORI Study
Source: Hypertension. 2026 Mar 6;83(6):e25431. doi: 10.1161/HYPERTENSIONAHA.125.25431 (PMC13189381; doi:10.1161/HYPERTENSIONAHA.125.25431)
Supplement: Supplementary file 1 [file hyp-83-e25431-s001.pdf]

A Randomized Study of Antithrombin in Early-Onset Preeclampsia: KOUNO-TORI Study

Jun TAKEDA, MD, PhD, Daisuke TACHIBANA, MD, PhD, Atsuo ITAKURA, MD, PhD, Kenichi TAKAGI, Shoichi NAKAMI, Hirotaka MANO, Takao KOBAYASHI, MD, PhD, Naohiro KANAYAMA, MD, Hiroshi SAMESHIMA, MD, PhD, Mamoru MORIKAWA, MD, PhD, Haruhiko SAGO, MD, PhD, Tomoko ADACHI, MD, PhD, Akihide OHKUCHI, MD, PhD, Satoru TAKEDA, MD, PhD, Hisashi MASUYAMA, MD, PhD, Hiroyuki SEKI, MD, PhD, Shigeru SAITO, MD, PhD
On behalf of the KOUNO-TORI Study Group

Corresponding author
Shigeru SAITO, MD, PhD, University of Toyama, 3190 Gofuku, Toyama 930-8555, Japan. Tel (work): +81-(0)76-445-6000, Email: s30saito@med.u-toyama.ac.jp

Supplemental Text

Supplemental methods

Diagnosis criteria of preeclampsia[1]

Preeclampsia was diagnosed when the women presented one or more of following clinical symptoms:

1. Gestational hypertension[a] occurring after 20 weeks of gestation, accompanied by proteinuria[b]. All symptoms were normalized by 12 weeks postpartum.
2. Hypertension occurring after 20 weeks of gestation, with or without proteinuria, but by one or more of the following new-onset conditions at or after 20 weeks of gestation. All symptoms were normalized by 12 weeks postpartum:
   - 2-1. Liver disorders indicated by elevated blood values of enzymes, such as aspartate aminotransferase or alanine transaminase >40 IU/L, without any underlying disease
   - 2-2. Progressive kidney injury indicated by elevated creatinine levels >1.0 mg/dL, without any other renal diseases.
   - 2-3. Blood coagulation disorders, such as thrombocytopenia (<150,000/μL) accompanied by hypertensive disorders of pregnancy, disseminated intravascular coagulation, or hemolysis.
3. Hypertension occurring after 22 weeks of gestation, accompanied by uteroplacental dysfunction (fetal growth restriction[c] or abnormal umbilical artery Doppler wave form analysis[d]), with or without proteinuria.[a] Hypertension is defined as having a systolic blood pressure ≥140 mmHg and/or diastolic blood pressure ≥90 mmHg

[a]Hypertension is defined as having a systolic blood pressure ≥140 mmHg and/or diastolic blood pressure ≥90 mmHg

[b]Proteinuria is diagnosed when 24-hour urinary protein is ≥300 mg per day by the Esbach method and/or a spot urinary protein/creatinine ratio is ≥0.3 mg/mg.

[c]Fetal growth restriction is clinically diagnosed when the estimated fetal body weight falls below mean weight minus 1.5 standard deviation for the gestational age based on the Japanese fetal growth curve developed by Okai,[2] without chromosomal abnormalities or malformation syndrome.

[d]Abnormal umbilical artery doppler wave form is assumed to be abnormally high umbilical arterial vascular resistance, end-diastolic blood flow disruption, or regurgitation.

Severity criteria for preeclampsia[3]

Preeclampsia accompanied by one or both conditions below was defined as severe.

1. Systolic blood pressure that is ≥160 mmHg and/or diastolic blood pressure that is ≥110 mmHg
2. Maternal organ involvement or uteroplacental dysfunction

Supplemental references

1. Japan Society for the Study of Hypertension in Pregnancy, eds. [Best Practice Guide 2021 for Care and Treatment of Hypertension in Pregnancy.] Tokyo: Medical View; 2021. In Japanese.
2. Okai T. [Standard Values of Ultrasonic Measurements in Japanese Fetuses]. *J Med Ultrason*. 2003;30:J415–J440. In Japanese.

3. Saito S, Takagi K, Moriya J, Kobayashi T, Kanayama N, Sameshima H, Morikawa M, Sago H, Adachi T, Ohkuchi A, et al. A Randomized Phase 3 Trial Evaluating Antithrombin Gamma Treatment in Japanese Patients with Early-Onset Severe Preeclampsia (KOUNO-TORI study): Study Protocol. *Contemp Clin Trials*. 2021;107:106490. doi: 10.1016/j.cct.2021.106490

Supplemental Tables

Table S1 Reasons for early delivery of pregnancy

Maternal indications

1. Uncontrolled high blood pressure (systolic blood pressure of ≥160 mmHg and/or diastolic blood pressure of ≥110 mmHg)
2. Decreased platelets (<100,000/mm3) or abnormal coagulation (rapid worsening within 6–12 hours)
3. Hepatic impairment (blood values more than twice the normal levels)
4. Persistent right hypochondrial pain or epigastric pain
5. Hemolysis, elevated liver enzymes, and low platelets (HELLP) syndrome
6. Progressive renal impairment (if no other renal disease is present, creatinine of ≥1.1 mg/dL or blood values more than twice the normal levels)
7. Lung edema
8. Severe pleural effusion, severe ascites, and serous retinal detachment
9. Central nervous system disorders (i.e., eclampsia, stroke) or abnormal vision (cortical blindness)
10. Severe headache and imminent eclampsia
11. Early placental abruption
12. Severe hypertensive disorders of pregnancy with severe hypertension in patients at ≥34 weeks of gestation

Fetal indication

1. Fetal placental dysfunction, non-reassuring fetal status, abnormal umbilical cord blood flow (i.e., regurgitation, persistent diastolic flow shutdown, or strict management), severe intrauterine growth restriction, arrest of fetal growth or fetal head circumference for ≥2 weeks, and oligohydramnios (amniotic fluid index of ≤5.0 cm and maximum vertical pocket of ≤2.0 cm)

Table S2. List of principal investigators, medical institutions, and the number of registered participants at each site

Principal investigator	Medical institution	N
Soromon Kataoka	Hakodate Central General Hospital	4
Hiroshi Asano	Hokkaido University Hospital	1
Rifumi Hattori	JA Obihiro Kosei Hospital	1
Emi Hirayama	Sapporo City General Hospital	1
Hisaya Suzuki	Japanese Red Cross Sendai Hospital	2
Katsuhiko Naruse	Dokkyo Medical University Hospital	1
Akihide Ohkuchi	Jichi Medical University Hospital	3
Akihiko Kikuchi	Saitama Medical University Hospital	8
Shintaro Makino	Juntendo University Urayasu Hospital	1
Masaki Ogawa	Tokyo Women's Medical University Yachiyo Medical Center	2
Yoshiharu Takeda	Aiiku Hospital	2
Michiko Kido	Japanese Red Cross Medical Center	2
Atsuo Itakura	Juntendo University Hospital	8
Shinji Tanigaki	Kyorin University Hospital	1
Haruhiko Sago	National Center for Child Health and Development	4
Mayumi Tokunaka	Showa University Hospital	1
Takayuki Iriyama	The University of Tokyo Hospital	5
Masahiko Nakata	Toho University Omori Medical Center	1
Hironobu Hyodo	Tokyo Metropolitan Bokutoh Hospital	2
Misako Iwata	Tokyo Metropolitan Ohtsuka Hospital	1
Yoshimi Taniguchi	Tokyo Metropolitan Tama Medical Center	1
Yoko Onishi	Kitasato University Hospital	1
Junichi Hasegawa	St. Marianna University School of Medicine Hospital	4
Shigeru Aoki	Yokohama City University Medical Center	3
Noriko Yoneda	Toyama University Hospital	4
Shinya Hirabuki	Ishikawa Prefectural Central Hospital	4
Yuzo Uchida	Yamanashi Prefectural Central Hospital	4
Norihiko Kikuchi	Shinshu University Hospital	1
Mariko Serizawa	Hamamatsu Medical Center	3
Toshitaka Tanaka	Juntendo University Shizuoka Hospital	2
Haruki Nishizawa	Fujita Health University Hospital	2
Hiroyuki Tsuda	Japanese Red Cross Aichi Medical Center Nagoya Daiichi Hospital	4
Yasuyuki Kishigami	Toyota Memorial Hospital	4
Hiroaki Tanaka	Mie University Hospital	4
Haruta Mogami	Kyoto University Hospital	1
Miyoko Waratani	University Hospital, Kyoto Prefectural University of Medicine	1

Mamoru Morikawa	Kansai Medical University	1
Jun Yoshimatsu	National Cerebral and Cardiovascular Center	1
Osamu Nakamoto	Osaka City General Hospital	9
Daisuke Tachibana	Osaka Metropolitan University Hospital	7
Keisuke Ishii	Osaka Women's and Children's Hospital	4
Kazuhide Ogita	Rinku General Medical Center	2
Kenji Tanimura	Kobe University Hospital	3
Fuminori Kimura	Nara Medical University Hospital	2
Toshiyuki Sado	Nara Prefecture General Medical Center	1
Hisashi Masuyama	Okayama University Hospital	2
Junichi Kodama	Hiroshima City Hospital	5
Susumu Murata	Yamaguchi University Hospital	1
Kazuhisa Maeda	National Hospital Organization: Shikoku Medical Center for Children and Adults	2
Yuka Uchikura	Ehime University Hospital	3
Masamitsu Kurakazu	Fukuoka University Hospital	6
Toshiyuki Yoshizato	Kurume University Hospital	4
Kosuke Kawakami	National Hospital Organization: Kokura Medical Center	4
Daizo Hori	St. Mary's Hospital	4
Kiyonori Miura	Nagasaki University Hospital	2
Sachie Suga	National Hospital Organization: Nagasaki Medical Center	6
Takashi Ohba	Kumamoto University Hospital	2
Kazuki Toyofuku	Oita Prefectural Hospital	3
Shinji Katsuragi	University of Miyazaki Hospital	3
Masato Kamitomo	Kagoshima City Hospital	4
Mikio Hashiguchi	Okinawa Prefectural Chubu Hospital	6

We gratefully acknowledge following investigators (institutions) for their participation in the study, but no patient was included at their sites: Takashi Ozaki (Aomori Prefectural Central Hospital); Tsukasa Baba (Iwate Medical University Hospital); Koji Nishijima (Niigata University Medical & Dental Hospital); Naoaki Tamura (Hamamatsu University Hospital); Tomoko Nakayama (Japanese Red Cross Society Himeji Hospital).

Table S3. Main reasons for delivery in the full analysis set

Main reason for delivery, n	Placebo, N = 91	rhAT-gamma, N = 90
Hypertension resistant to therapy	15	12
Thrombocytopenia/coagulation abnormalities	6	2
Hepatic function disorder	8	2
Persistent right hypochondoralgia, epigastric pain	1	1
HELLP syndrome	0	0
Advanced renal insufficiency	11	5
Pulmonary edema	1	2
Severe pleural effusion, severe ascites, and serous retinal detachment	9	11
CNS involvement or visual abnormalities	1	0
Severe headache, impending eclampsia	0	1
Abruptio placentae	0	1
After 34-week gestation in a severe hypertensive disorder of pregnancy with severe hypertension	3	5
Fetoplacental insufficiency	29	35
Other	7	13

CNS, central nervous system; HELLP syndrome, hemolysis, elevated liver enzymes, and low platelets syndrome; rhAT, recombinant human antithrombin

Table S4. Summary of maternal, fetal, and neonatal efficacy endpoints in full analysis set

Parameter	Placebo N = 91	rhAT-gamma N = 90	Mean difference: rhAT-gamma − placebo (95% CI)
Maternal			
Delivery style			
n	90	89	
Cesarean section, n (%)	87 (96.7)	81 (91.0)	−5.7 (−20.0, 9.0)
Pregnancy termination during treatment period			
n	90	90	
Yes (%)	35 (38.9)	26 (28.9)	−10.0 (−24.8, 5.2)
Blood loss during delivery, mL			
n	90	89	
Mean ± SD	784.7 ± 548.9	806.5 ± 581.9	21.8 (−145.0, 188.7)
Fetal			
Fetal growth rate, g/day			
Day 4			
n	74	75	
Mean ± SD	19.1 ± 28.4	19.2 ± 25.3	0.1 (−8.6, 8.8)
Day 8			
n	49	57	
Mean ± SD	15.41 ± 9.864	15.67 ± 10.829	0.3 (−3.8, 4.3)
Biophysical profile score			
Day 4			
n	71	71	
Mean±SD	9.4±1.32	9.7 ± 0.70	0.3 (−0.1, 0.6)
Day 8			
n	50	58	

Parameter	Placebo<br>N = 91	rhAT-gamma<br>N = 90	Mean difference: rhAT-gamma − placebo (95% CI)
Mean ± SD	9.5 ± 0.97	9.3 ± 1.69	−0.1 (−0.7, 0.4)
Neonatal			
Birth weight, g			
n	90	89	
Mean ± SD	1101.2 ± 397.4	1217.4 ± 504.3	116.2 (−17.6, 250.0)
Birth height, cm			
n	78	85	
Mean ± SD	36.9 ± 4.0	37.4 ± 4.6	0.5 (−0.9, 1.8)
NICU hospitalization			
n	91	90	
Yes, n (%)	89 (97.8)	87 (96.7)	−1.1 (−15.57, 13.39)
Duration of NICU hospitalization, days			
n	89	87	
Mean ± SD	50.8 ± 33.6	45.4 ± 34.1	−5.4 (−15.5, 4.6)
Respiratory management			
n	91	90	
Present, n (%)	83 (91.2)	79 (87.8)	−3.4 (−17.74, 11.21)
Gas*, n (%)	32 (38.6)	33 (41.8)	-
Noninvasive respiratory therapy, n (%)	64 (77.1)	61 (77.2)	-
Respiratory management with a ventilator, n (%)	47 (56.6)	44 (55.7)	-
Other, n (%)	0 (0.0)	0 (0.0)	-
Duration of respiratory management, days			
n	83	79	

Parameter	Placebo<br>N = 91	rhAT-gamma<br>N = 90	Mean difference: rhAT-gamma − placebo (95% CI)
Mean ± SD	35.6 ± 29.2	30.5 ± 28.0	−5.0 (−13.9, 3.9)
Size of the neonate, n (%)			
n	90	89	
Small for gestational age, n (%)	78 (86.7)	67 (75.3)	-
Appropriate for gestational age, n (%)	12 (13.3)	22 (24.7)	-
Large for gestational age, n (%)	0 (0.0)	0 (0.0)	-
Bronchopulmonary dysplasia*			
n	91	90	
Yes, n (%)	4 (4.4)	7 (7.8)	3.4 (−11.2, 17.7)
Intraventricular hemorrhage†			
n	91	90	
Yes, n (%)	4 (4.4)	4 (4.4)	0.0 (−14.5, 14.5)
Periventricular leukomalacia†			
n	91	90	
Yes, n (%)	0 (0.0)	1 (1.1)	1.1 (−13.4, 15.6)
Retinopathy of prematurity†			
n	91	90	
Yes, n (%)	3 (3.3)	3 (3.3)	0.0 (−14.5, 14.5)
Sepsis†			
n	91	90	
Yes, n (%)	1 (1.1)	4 (4.4)	3.3 (−11.21, 17.74)

Parameter	Placebo N = 91	rhAT-gamma N = 90	Mean difference: rhAT-gamma − placebo (95% CI)
Necrotizing enteritis†			
n	91	90	
Yes, n (%)	0 (0.0)	0 (0.0)	0.0 (-, -)
Death†			
n	91	90	
Yes, n (%)	0 (0.0)	3 (3.3)	3.3 (−11.21, 17.74)

*Gases include supplementary oxygen, nitrous oxide, and others

†Short-term prognosis for 28 days after delivery

CI, confidence interval; NICU, neonatal intensive care unit; rhAT, recombinant human antithrombin; SD, standard deviation

Table S5. Hazard ratio for pregnancy prolongation by Cox proportional-hazard model

Covariate	Hazard ratio (95% confidence interval)
Treatment	
Placebo	Reference
rhAT-gamma	0.774 (0.577, 1.040)
Baseline AT activity	
≤80%	Reference
>80%	0.670 (0.496, 0.906)
Gestational age	
<28 weeks	Reference
≥28 weeks	1.024 (0.742, 1.412)
Proteinuria	
Present	Reference
Absent	0.541 (0.346, 0.847)

AT, antithrombin; rhAT, recombinant human antithrombin

Table S6. Summary of achieved gestational age in full analysis set

	Placebo, N = 91		rhAT-gamma, N = 90		
Achievement of gestation, weeks	n (%)	95% CI	n (%)	95% CI	Difference: rhAT-gamma − placebo, % (95% CI)
28 weeks					
n	30		31		
Achieved	15 (50.0)	31.3, 68.7	19 (61.3)	42.2, 78.2	11.3 (−14.8, 35.8)
32 weeks					
n	91		90		
Achieved	29 (31.9)	22.5, 42.5	35 (38.9)	28.8, 49.7	7.0 (−7.9, 21.0)
34 weeks					
n	91		90		
Achieved	12 (13.2)	7.0, 21.9	17 (18.9)	11.4, 28.5	5.7 (−9.0, 19.9)

CI, confidence interval; rhAT, recombinant human antithrombin

Table S7. Summary of other biomarkers in full analysis set

	Placebo, N = 91			rhAT-gamma, N = 90		
Parameter	Observed values	Change from baseline	Percent change from baseline	Observed values	Change from baseline	Percent change from baseline
sFlt-1, pg/mL						
Baseline						
n	91	-	-	90	-	-
Mean ± SD	13606.9 ± 8394.1	-	-	12525.0 ± 6161.6	-	-
Day 4						
n	77	77	77	74	74	74
Mean ± SD	15703.9 ± 11361.0	2574.9 ± 4313.8	20.0 ± 29.5	13265.4 ± 6029.5	1632.4 ± 3839.4	18.8 ± 36.8
Day 8						
n	53	53	53	58	58	58
Mean ± SD	14830.6 ± 11945.1	2960.6 ± 4486.3	27.87 ± 34.3	13122.4 ± 6436.9	1955.4 ± 3738.1	21.3 ± 32.9
PlGF, pg/mL						
Baseline						
n	90	-	-	90	-	-
Mean ± SD	40.2 ± 28.6	-	-	60.0 ± 95.4	-	-
Day 4						
n	77	76	76	74	74	74

	Placebo, N = 91			rhAT-gamma, N = 90		
Parameter	Observed values	Change from baseline	Percent change from baseline	Observed values	Change from baseline	Percent change from baseline
Mean ± SD	38.2 ± 26.3	−3.7 ± 14.6	−4.1 ± 30.6	53.7 ± 69.0	−9.9 ± 43.3	−1.1 ± 33.5
Day 8						
n	53	52	52	58	58	58
Mean ± SD	41.2 ± 25.8	−4.9 ± 15.1	−3.6 ± 35.5	58.2 ± 76.8	−12.3 ± 48.6	−4.3 ± 28.5
sFlt-1/PlGF ratio						
Baseline						
n	90	-	-	90	-	-
Mean ± SD	517.1 ± 458.2	-	-	465.3 ± 448.8	-	-
Day 4						
n	77	76	76	74	74	74
Mean ± SD	652.1 ± 649.9	168.1 ± 308.5	39.5 ± 68.6	463.7 ± 402.4	28.6779 ± 244.5	34.3 ± 61.3
Day 8						
n	53	52	52	58	58	58
Mean ± SD	507.1 ± 408.2	125.0 ± 221.9	51.7 ± 85.3	487.2 ± 463.4	60.4 ± 246.7	44.4 ± 73.9

PlGF, placental growth factor; rhAT, recombinant human antithrombin; SD, standard deviation; sFlt-1, soluble fms-like tyrosine kinase-1

Table S8. Incidence of adverse events of special interest in safety analysis set

Category	Placebo<br>N = 91<br>n (%)	rhAT-gamma<br>N = 90<br>n (%)	Difference: rhAT-gamma – placebo<br>% (95% CI)
Patients with any adverse events of special interest	8 (8.8)	25 (27.8)	19.0 (4.3, 32,7)
Hemorrhage-related adverse events	8 (8.8)	25 (27.8)	19.0 (4.3, 32,7)
Hemorrhage and hematoma	8 (8.8)	24 (26.7)	17.9 (3.1, 31.7)
Epistaxis	1 (1.1)	3 (3.3)	-
Uterine hemorrhage	1 (1.1)	3 (3.3)	-
Procedural hemorrhage	2 (2.2)	2 (2.2)	-
Hemorrhage subcutaneous	1 (1.1)	2 (2.2)	-
Premature separation of placenta	1 (1.1)	2 (2.2)	-
Post-procedural hemorrhage	1 (1.1)	2 (2.2)	
Subcutaneous hematoma	0 (0.0)	2 (2.2)	-
Abdominal wall hemorrhage	0 (0.0)	2 (2.2)	-
Cerebral hemorrhage	0 (0.0)	1 (1.1)	-
Extradural hematoma	0 (0.0)	1 (1.1)	-
Hematoma	0 (0.0)	1 (1.1)	-
Pelvic hematoma, obstetric	1 (1.1)	0 (0.0)	-
Postpartum hemorrhage	0 (0.0)	1 (1.1)	-
Cervix hematoma, uterine	0 (0.0)	1 (1.1)	-
Incision site hemorrhage	1 (1.1)	0 (0.0)	-
Wound hemorrhage	0 (0.0)	1 (1.1)	-
Hematoma, muscle	0 (0.0)	1 (1.1)	-
Abdominal wall hematoma	0 (0.0)	1 (1.1)	-
Intrapartum hemorrhage	0 (0.0)	1 (1.1)	-
Wound hematoma	0 (0.0)	1 (1.1)	-
Others	0 (0.0)	5 (5.6)	

Category	Placebo<br>N = 91<br>n (%)	rhAT-gamma<br>N = 90<br>n (%)	Difference: rhAT-gamma – placebo<br>% (95% CI)
Purpura	0 (0.0)	2 (2.2)	2.2 (−12.3, 16.7)
Disseminated intravascular coagulation	0 (0.0)	2 (2.2)	2.2 (−12.3, 16.7)
Immune thrombocytopenia	0 (0.0)	1 (1.1)	1.1 (−13.4, 15.6)

CI, confidence interval; rhAT, recombinant human antithrombin

Table S9. Incidence of drug-related TEAEs in safety analysis set

Category	Placebo N = 91 n (%)	rhAT-gamma N = 90 n (%)
Maternal		
Patients with any drug-related TEAE	2 (2.2)	7 (7.8)
Gastrointestinal disorders	0 (0.0)	1 (1.1)
Abdominal wall hemorrhage	0 (0.0)	1 (1.1)
General disorders and administration site conditions	0 (0.0)	1 (1.1)
Peripheral swelling	0 (0.0)	1 (1.1)
Injury, poisoning, and procedural complications	1 (1.1)	0 (0.0)
Post-procedural hemorrhage	1 (1.1)	0 (0.0)
Metabolism and nutritional disorders	1 (1.1)	1 (1.1)
Hypertriglyceridemia	1 (1.1)	1 (1.1)
Nervous system disorders	0 (0.0)	1 (1.1)
Headache	0 (0.0)	1 (1.1)
Pregnancy, puerperium, and perinatal conditions	0 (0.0)	1 (1.1)
Postpartum hemorrhage	0 (0.0)	1 (1.1)
Respiratory, thoracic, and mediastinal disorders	0 (0.0)	1 (1.1)
Epistaxis	0 (0.0)	1 (1.1)
Skin and subcutaneous tissue disorders	0 (0.0)	1 (1.1)
Rash	0 (0.0)	1 (1.1)
Vascular disorders	0 (0.0)	1 (1.1)
Vascular pain	0 (0.0)	1 (1.1)
Fetal		
Any drug-related TEAE	0 (0.0)	0 (0.0)
Neonatal		
Any drug-related TEAE	0 (0.0)	1 (1.1)
Respiratory, thoracic, and mediastinal disorders	0 (0.0)	1 (1.1)
Neonatal pulmonary hypertension	0 (0.0)	1 (1.1)

rhAT, recombinant human antithrombin; TEAE, treatment-emergent adverse event
These AEs were diagnosed by the investigator and reported at the investigator's discretion.

Table S10. Incidence of hemorrhage-related adverse events (adverse events of special interest) and anemia by timing of delivery in safety analysis set

Event	Placebo, N = 91				rhAT-gamma, N = 90			
	Before delivery		Day of delivery, n (%)	After delivery, n (%)	Before delivery		Day of delivery, n (%)	After delivery, n (%)
	During treatment, n (%)	After treatment, n (%)			During treatment, n (%)	After treatment, n (%)		
n	91	54	88	88	90	62	85	85
Hemorrhage-related adverse events	1 (1.1)*	0 (0.0)	5 (5.7)	3 (3.4)	4 (4.4)†	3 (4.8)‡	10 (11.8)	14 (16.5)
Anemia	2 (2.2)	0 (0.0)	3 (3.4)	4 (4.5)	1 (1.1)	2 (3.2)	6 (7.1)	15 (17.6)

*Epistaxis (n=1)
†Epistaxis (n=2), cervix hematoma uterine (n=1), and purpura (n=1)
‡Epistaxis (n=1), hemorrhage subcutaneous (n=1), immune thrombocytopenia (n=1)
rhAT, recombinant human antithrombin

Supplemental Figures

Figure S1. Kaplan–Meier plot of pregnancy prolongation in per-protocol set

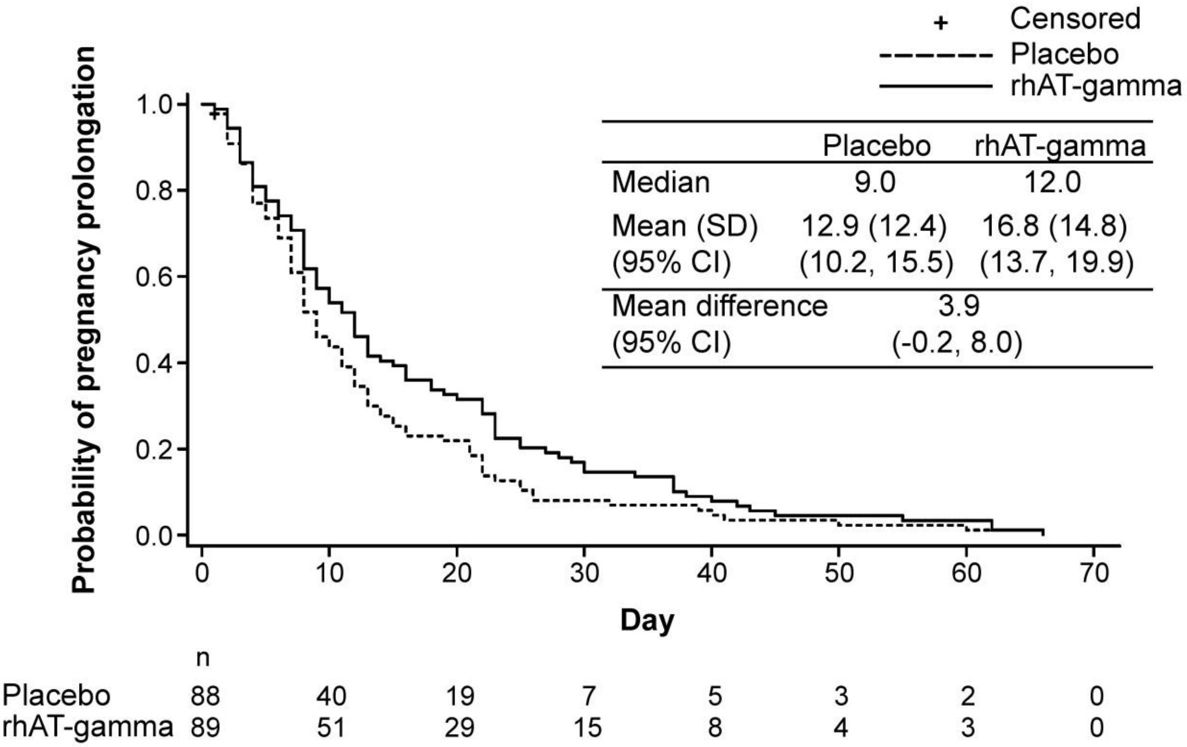

This Kaplan–Meier plot shows the pregnancy prolongation period for the per-protocol set in the rhAT-gamma group and placebo group. The solid line represents the rhAT-gamma group, the dotted line represents the placebo group, and a plus sign represents a censored patient.

CI, confidence interval; rhAT, recombinant human antithrombin; SD, standard deviation

Major Resources Table

In order to allow validation and replication of experiments, all essential research materials listed in the Methods should be included in the Major Resources Table below. Authors are encouraged to use public repositories for protocols, data, code, and other materials and provide persistent identifiers and/or links to repositories when available. Authors may add or delete rows as needed.

Animals (in vivo studies)

Species	Vendor or Source	Background Strain	Sex	Persistent ID / URL
–	–	–	–	–
–	–	–	–	–
–	–	–	–	–

Genetically Modified Animals

	Species	Vendor or Source	Background Strain	Other Information	Persistent ID / URL
**Parent - Male**	–	–	–	–	–
**Parent - Female**	–	–	–	–	–

Antibodies

Target antigen	Vendor or Source	Catalog #	Working concentration	Lot # (preferred but not required)	Persistent ID / URL
–	–	–	–	–	–
–	–	–	–	–	–

DNA/cDNA Clones

Clone Name	Sequence	Source / Repository	Persistent ID / URL
–	–	–	–
–	–	–	–
–	–	–	–

Cultured Cells

Name	Vendor or Source	Sex (F, M, or unknown)	Persistent ID / URL
–	–	–	–
–	–	–	–
–	–	–	–

Data & Code Availability

Description	Source / Repository	Persistent ID / URL
–	–	–
–	–	–
–	–	–

Other

Description	Source / Repository	Persistent ID / URL
Protocol	jRCT	https://jrct.mhlw.go.jp/ (jRCT2080224912)
Statistical Analysis Plan	jRCT	https://jrct.mhlw.go.jp/ (jRCT2080224912)
